# Pharmacoeconomic analysis of biologics and methotrexate for rheumatoid arthritis from the standpoint of the number needed to treat concept under the Japanese health insurance system

**DOI:** 10.1186/s12962-022-00347-2

**Published:** 2022-03-24

**Authors:** Kengo Harigane, Yuichi Mochida, Takayuki Shimazaki, Naomi Kobayashi, Yutaka Inaba

**Affiliations:** 1 Center for Rheumatic Diseases, Yokohama City University Medical Center, 4-57 Urafune-cho, Minami-ku, Yokohama, Kanagawa Japan; 2 Department of Orthopaedic Surgery, Yokohama City University Medical Center, Yokohama, Japan; 3 Department of Orthopaedic Surgery, Yokohama City University School of Medicine, Yokohama, Japan

**Keywords:** Biologics, Cost-effectiveness, Methotrexate, Number needed to treat, Rheumatoid arthritis

## Abstract

**Objectives:**

To evaluate the cost-effectiveness of biologics and methotrexate (MTX) for rheumatoid arthritis (RA) using the number needed to treat (NNT) concept and total actual health care cost.

**Methods:**

This study included 121 RA patients with newly prescribed biologics and/or MTX between 2012 and 2017. The NNT was calculated based on the 24 week remission rate of Disease Activity Score in 28 joints using erythrocyte sedimentation rate (DAS28-ESR) and Clinical Disease Activity Index (CDAI).

**Results:**

Remission rates were 76.4% for DAS28-ESR and 45.4% for CDAI in the biologics group and 63.6% and 24.2%, respectively, in the MTX group. The NNT was calculated as 6.4 and 4.2 in the biologics group and 34.2 and 35.2 in the MTX group, respectively. Mean total actual health care costs were 1,044,066 JPY (9835 US$)/24 weeks per treated patient in the biologics group and 75,860 JPY (715 US$)/24 weeks in the MTX group. Although the effectiveness of biologics was superior to MTX from the standpoint of NNT, the mean total health care cost and mean cost per NNT were much higher in the biologics group.

**Conclusions:**

Cost-effectiveness is clearly higher for MTX than biologics from the standpoint of mean total health care cost per adjusted NNT under the Japanese health insurance system.

## Introduction

Biologic disease-modifying anti-rheumatic drugs (bDMARDs) have recently become widely used for patients with rheumatoid arthritis (RA). Although more expensive, they are reported to have higher effectiveness than other disease-modifying anti-rheumatic drugs (DMARDs), including methotrexate (MTX) [1–4]. Because RA is a chronic inflammatory disease and the treatment usually extends over a long period of time, evaluation of the cost-effectiveness of biologics is important. Smolen et al. [5] in the 2019 European League Against Rheumatism (EULAR) recommendations for the management of RA stated that RA incurs high individual, medical, and societal costs, all of which should be considered in its management by the treating rheumatologist. However, it is relatively difficult to calculate the actual costs for many patients, so there are few comparative studies of actual costs and the cost-effectiveness of bDMARDs and MTX.

The number needed to treat (NNT) is an indicator that represents the effectiveness of therapeutic drugs [6, 7]. It is an index for determining the number of patients who need to be treated in order for one patient to achieve a clinical goal when a new treatment or drug is introduced.

$${\text{NNT}} = 1/{\text{ARR}}\,\left( {{\text{absolute}}\,{\text{risk}}\,{\text{reduction}}} \right).$$

$${\text{ARR}} = \left( {{\text{the}}\,{\text{remission}}\,{\text{rate}}\,{\text{of}}\,{\text{bDMARDs}}/{\text{MTX}}\,{\text{group}}} \right) - \left( {{\text{the}}\,{\text{remission}}\,{\text{rate}}\,{\text{of}}\,{\text{control}}\,{\text{group}}} \right).$$

Although the NNT is not itself a pharmacoeconomic indicator, some studies have reported the cost-effectiveness of bDMARDs for RA treatment from the standpoint of NNT and the relevant costs per NNT. Batticciotto et al. [8] reported that tocilizumab (TCZ) was a more cost-effective option than tumor necrosis factor-α (TNF-α) inhibitors as first-line monotherapy for patients intolerant to MTX. Benucci et al. [9] also reported TCZ as a cost-effective option compared with abatacept (ABT) in patients previously treated with MTX. Although these two studies compared bDMARDs, the relevant costs of treatment and monitoring activities were calculated based on simulations from previous studies. The total health care cost estimated based on simulations in these study does not include the cost of additional costs such as regular monitoring of chest X-ray for the patients with interstitial pneumonia or blood examinations of deoxyribonucleic acid of hepatitis B virus (HBV) for the patients who had prior infection with HBV. Therefore, they may have underestimated the actual costs. The actual total health care costs investigated in this study included these additional costs which was needed for the management of RA. To our knowledge, no studies to date have investigated the actual costs of bDMARDs and to compare the cost-effectiveness of bDMARDs with that of MTX in patients with RA. It is therefore important to investigate the actual costs, not based on the simulation, and compare the cost- effectiveness of bDMARDs with MTX in the real-world clinical setting.

The aims of this study were to evaluate the cost-effectiveness of bDMARDs and MTX in patients with RA, using NNT, in addition, the total actual costs were investigated and compared between bDMARDs and MTX.

## Materials and methods

### Patients

**Fig. 1:**
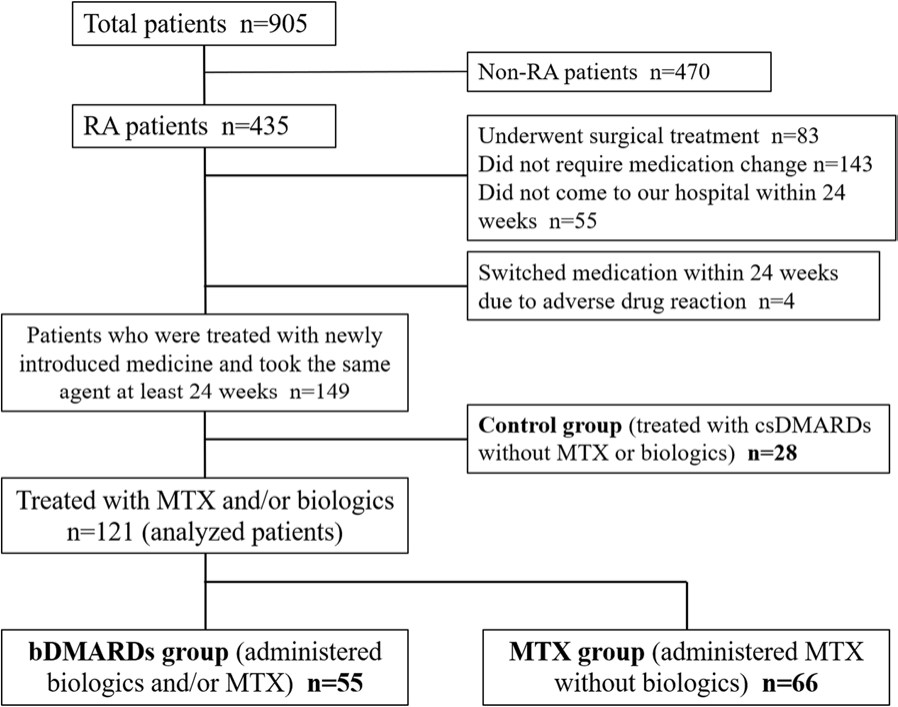
Flowchart of patient enrollment in this study. In total, 905 consecutive patients newly presented to our hospital between 2012 and 2017. After excluding 756 patients, we enrolled and analyzed the remaining 149 RA patients. Patients divided into a bDMARDs group (55 patients), MTX group (66 patients), or control group (28 patients)

The study flowchart is shown in Fig. 1. In total, 905 consecutive patients newly presented to our hospital between September 2012 and March 2017. Of them, 470 diagnosed as not having RA were excluded from this study. Among the 435 patients with RA, those who underwent surgical treatment, did not need to change medications, or did not visit our hospital within 24 weeks (lost to follow-up or failed to return to hospital or clinic) were also excluded. Of 153 patients treated with newly introduced medication, 4 patients who needed to switch medication within 24 weeks due to an adverse drug reaction were excluded in line with previous studies [8, 9]. Twenty-eight patients treated with conventional synthetic DMARDS (csDMARDs) without bDMARDs or MTX were used as control group to calculate the NNT. Ultimately, 121 patients treated with bDMARDs and/or MTX who continued with the same agent for at least 24 weeks were investigated and analyzed in this study. The patients were divided into a bDMARDs group (administered bDMARDs and/or MTX; 55 patients) or an MTX group (administered MTX without bDMARDs; 66 patients). Because this study was based on the real-world clinical setting, not randomized control trial, the selection of therapeutic drugs was decided by the attending physician. The patients in both groups were permitted combination therapy with csDMARDs or glucocorticoid. Remission rates for the Disease Activity Score in 28 joints using erythrocyte sedimentation rate (DAS28-ESR) and the Clinical Disease Activity Index (CDAI) at 24 weeks were retrospectively investigated. All patients fulfilled the 1987 American.

College of Rheumatology (ACR) classification criteria for RA [10] and/or the 2010 ACR/EULAR classification criteria for RA [11].

This study was conducted with approval from Ethics Committee (Approval Number: B190300015). Written informed consent to participate and publish the material was obtained from all patients included in the study.

### NNT

As previously mentioned, NNT is not itself a pharmacoeconomic indicator but is an indicator of the effectiveness of therapeutic drug or treatment intervention. It is an index for determining the number of patients who need to be treated in order for one patient to achieve a clinical goal when a new drug or treatment intervention is introduced. Thus, the lower the NNT, the better the effectiveness of a drug or treatment intervention. In this study, we set remission on DAS28-ESR or CDAI at 24 weeks as the clinical goal. NNT is calculated as a reciprocal number of the absolute risk reduction (ARR). The ARR refers to the difference in the remission rate between the therapeutic group (bDMARDs group or MTX group) and the control group.

### Adjusted NNT

In this study, patients’ background including disease duration, Steinbrocker stage and class [12], and rate of MTX administration at the time of drug introduction were highly variable between the bDMARDs and MTX groups. Because the remission is critical in this type of study, the differences in the baseline data are very important. It could therefore be inappropriate to simply compare the NNT for each group, therefore based on the recommendations of a biostatistician, we excluded bias from differences in patient backgrounds between the bDMARDs and MTX groups and then calculated an adjusted NNT. The adjusted NNT was calculated with adjustment for confounding variables using the adjusted odds ratio (OR) estimated from multiple logistic regression. Logistic regression analysis was performed using DAS28-ESR or CDAI remission rates as an objective variable and patients’ background factors, such as age, sex, Steinbrocker stage, disease activity, and disease duration, as explanatory variables. Then, using these ORs, we estimated the number of remissions in the bDMARDs and MTX groups.

### Total health care cost under the Japanese health insurance system

For each patient, the actual total health care costs between the first visit and 24 weeks after the introduction of the therapeutic drugs, including the consultation fee, laboratory test fee, prescription fee, and cost of bDMARDs, were calculated as shown in the medical receipt and then averaged and compared between the groups. All patients underwent blood examination and chest radiography or computed tomography as screening tests immediately before the introduction of the bDMARDs or MTX. Therefore, the total actual costs in this study included the costs of these screening tests.

The aim of this study was to compare cost-effectiveness of bDMARDs with MTX. As previously mentioned, because bDMARDs are remarkably more expensive than MTX, simple comparison of total health care cost of bDMARDs with MTX is inadequate. Therefore, we investigated mean cost per NNT as an indicator of cost-effectiveness of bDMARDs and MTX. Mean cost per NNT represents the costs required for a single patient to achieve remission. In other words, it was calculated by multiplying the total actual health care costs by the NNT. The costs needed to treat adverse drug reactions or complications such as bacterial pneumonia or liver dysfunction were excluded from the amount in the medical receipt, in line with previous studies [8, 9].

### Time horizon

When two or more health care technologies are being compared for pharmacoeconomic purposes, international guidelines recommend the use of a suitable time horizon to delineate all the main differences expressed in terms of both outcomes and treatment costs [13]. The costs other than drug cost such as the initial costs of screening tests and the costs of regular visit fee and laboratory examinations after introduction of drugs were same in bDMARDs MTX groups. Therefore, a 24 week period was considered adequate to delineate the most important differences in effectiveness and treatment costs because the median follow-up effectiveness period is 24 weeks in many of the clinical studies considered in the National Institute for Health and Care Excellence guidelines [14].

### Statistical methods

Statistical analyses, except logistic regression analysis, were performed using BellCurve for Excel (ver. 2.15; Social Survey Research Information Co., Ltd., Tokyo, Japan). Logistic regression analysis was performed using EZR on R Commander version 1.31 (Saitama Medical Center, Jichi Medical University, Saitama, Japan). Kruskal–Wallis test was used to compare demographic data of the bDMARDs, MTX and control groups. Student’ t-test was used to compare changes in disease activity between the bDMARDs and MTX groups. Logistic regression analysis was applied to calculate the adjusted OR of the bDMARDs and MTX groups. p < 0.05 was considered statistically significant.

## Results

### Patient characteristics

The demographic and baseline clinical data of the bDMARDs, MTX, and control groups are shown in Table 1. There were significant differences in the mean disease duration, CDAI, Steinbrocker stage and class, and administration rates of MTX, salazosulfapyridine (SASP) and bucillamine (BUC) among three groups. Mean disease duration was significantly longer and Steinbrocker stage and class were significantly higher in the bDMARDs group than in the MTX group (both p < 0.01). Administration rates of salazosulfapyridine and bucillamine were lower in the bDMARDs group than in the MTX group (both p < 0.05). There were no significant differences in DAS28-ESR and CDAI between the bDMARDs and MTX groups at baseline.

**Table 1:** Demographic and baseline clinical data

	bDMARDS group (n = 55)	MTX group (n = 66)	Control group (n = 28)	p value
Age, years	53.5	57.0	64.4	n.s
Female sex (%)	70.1	75.8	75.0	n.s
Disease duration, years	5.8** ^††^	1.8	0.9	< 0.01
DAS28-ESR at baseline	4.36	3.96	3.75	n.s
CDAI at baseline	17.4^**^	15.9**	11.6	< 0.01
Steinbrocker stage	** ^††^			< 0.01
I	22	56	19	
II	17	5	9	
III	9	4	0	
IV	7	1	0	
Steinbrocker class	** ^††^			< 0.01
1	17	44	19	
2	30	22	9	
3	7	0	0	
4	1	0	0	
MTX (%)	76.3**	100**	0	< 0.01
MTX dose (mg/week)	6.8	7.2	0	n.s
Glucocorticoid (%)	40.0	24.2	25.0	n.s
SASP (%)	41.8*^†^	66.7	75.0	< 0.01
BUC (%)	23.6*^††^	59.1	53.6	< 0.01
IGU (%)	14.7	12.1	25.0	n.s
TAC (%)	5.4	0.0	3.6	n.s

bDMARDs group	55 cases			
TCZ	20			
GLM	16			
ETN	13			
CZP	3			
ABT	2			
IFX	1			

*bDMARDS* biologic disease-modifying anti-rheumatic drugs, *ABT* abatacept, *BUC* bucillamine, *CDAI* clinical disease activity index, *CZP* certolizumab pegol, *DAS28-ESR* disease activity score in 28 joints using erythrocyte sedimentation rate, *ETN* etanercept, *GLM* golimumab, *IFX* infliximab, *IGU* iguratimod, *MTX* methotrexate, *SASP* salazosulfapyridine, *TAC* tacrolimus, *TCZ* tocilizumab

^*^p < 0.05 compared with control group; ^**^p < 0.01 compared with control group

^†^p < 0.05 compared with MTX group; ††p < 0.01 compared with MTX group

In the bDMARDs group, TCZ was used in 20 patients, golimumab (GLM) in 16, etanercept (ETN) in 13, and other bDMARDs in 6 patients (Table 1). In the patients administered TCZ, 12 patients used a subcutaneous agent (162 mg/2 weeks) and 8 patients used an intravenous agent (8 mg/kg/4 weeks). In the case of GLM, 11 patients were administered a dosage of 100 mg/4 weeks and 5 patients were administered 50 mg/4 weeks. Among the 11 patients administered 100 mg of GLM, 8 patients continued to receive 100 mg for 24 weeks, 2 patients changed to 50 mg after 16 weeks, and 1 patient discontinued it at 12 weeks due to ineffectiveness. In the 5 patients administered 50 mg/4 weeks of GLM, the dosage was increased to 100 mg at 12 weeks and discontinued it at 16 weeks due to ineffectiveness in 1 patient. For ETN, all patients were administered 50 mg/week.

### Changes in disease activity

**Fig. 2:**
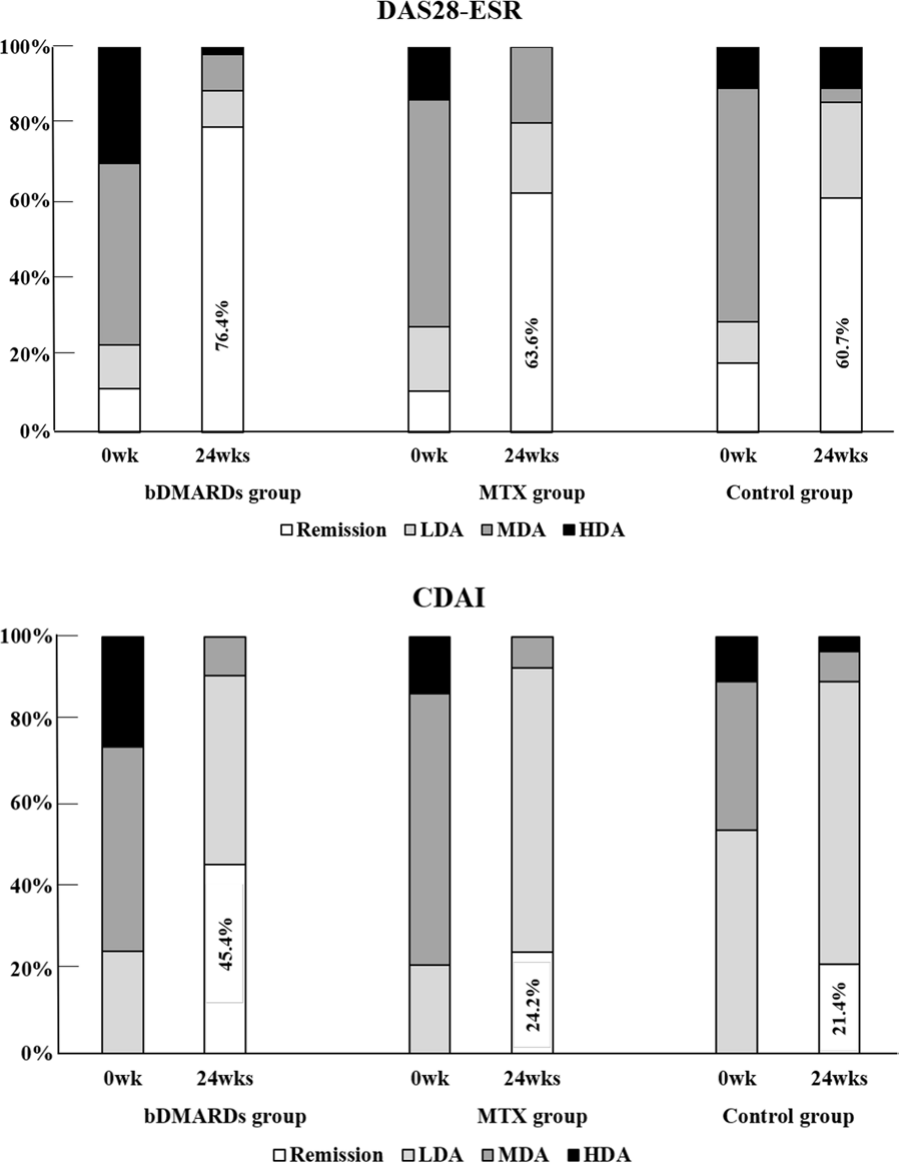
Changes in DAS28-ESR and CDAI between baseline and 24 weeks. Remission rates according to DAS28-ESR were 76.4% in the bDMARDs, 63.6% in MTX, and 60.7% in the control group, respectively (above). Remission rates at 24 wee1ks according to CDAI were 45.4% in the bDMARDs, 24.2% in MTX, and 21.4% in the control group, respectively (below)

At 24 weeks in the bDMARDs group, 42 patients (76.4%) achieved remission according to DAS28-ESR while 25 patients (45.4%) achieved remission according to CDAI. The corresponding rates were 63.6% and 24.2% in the MTX group and 60.7% and 21.4% in the control group (Fig. 2).

The mean DAS28-ESR significantly decreased in the bDMARDs group from 4.36 at baseline to 1.92 at 24 weeks (p < 0.001), and in the MTX group from 3.75 at baseline to 2.29 at 24 weeks (p < 0.001). There were significant differences in CDAI between baseline and 24 weeks in either the bDMARDs or the MTX group (bDMARDs group: 17.47 to 4.31, MTX group: 15.90 to 5.19, both p < 0.01, Table 2).

**Table 2:** Changes in DAS28-ESR, CDAI, NNT, total health care cost, and cost per NNT

	N	DAS28-ESR		Patient number of remissions	Remission rate	ARR	NNT	Mean total health care costs^a^ (mean ± SD)	Mean costs per NNT^a^
		Baseline	24 weeks						
bDMARDs	55	4.36	1.92	42	0.764	0.157	6.380	9834 ± 3808	62,741
TCZ	20	4.50	1.57	18	0.900	0.293	3.415	6926 ± 1452	23,652
GLM	16	4.11	2.70	10	0.625	0.018	55.556	15,034 ± 2696	835,229
ETN	13	4.35	2.05	10	0.769	0.162	6.169	8111 ± 1004	50,036
Others	6	4.62	2.38	4	0.667	0.060	16.800	10,388 ± 1587	174,518
MTX	66	3.75	2.29	42	0.636	0.029	34.222	715 ± 155	24,469

	N	CDAI		Patient number of remissions	Remission rate	ARR	NNT	Mean total health care costs^a^ (mean ± SD)	Mean costs per NNT^a^
		Baseline	24 weeks						
bDMARDs	55	17.47	4.31	25	0.454	0.241	4.157	9834 ± 3808	40,880
TCZ	20	19.00	2.94	11	0.550	0.336	2.979	6926 ± 1452	20,633
GLM	16	15.23	5.10	5	0.313	0.099	10.101	15,034 ± 2696	151,858
ETN	13	18.30	4.52	7	0.538	0.324	3.085	8111 ± 1004	25,022
Others	6	15.33	6.58	2	0.333	0.119	8.400	10,388 ± 1587	87,259
MTX	66	15.90	5.19	16	0.242	0.028	35.538	715 ± 155	25,410

*bDMARDS* biologic disease-modifying anti-rheumatic drugs, *ARR* absolute risk reduction, *CDAI* clinical disease activity index, *DAS28-ESR* disease activity score in 28 joints using erythrocyte sedimentation rate, *ETN* etanercept, *GLM* golimumab, *MTX* methotrexate, *SD* standard deviation, *NNT* number needed to treat, *TCZ* tocilizumab

^a^US$/24 weeks/patient

### NNT

NNTs were calculated as 6.38 based on DAS28-ESR and 4.16 based on CDAI in the bDMARDs group and 34.22 and 35.54, respectively, in the MTX group (Table 2). NNT of bDMARDs was approximately fivefold superior than that of MTX based on DAS28-ESR or eightfold superior based on CDAI. In the bDMARDs group, although NNTs based on both DAS28-ESR and CDAI in TCZ and ETN patients were lower than those of the other agents, there was no significant difference among the biologic agents.

### Total actual health care cost

The mean total actual health care costs were 1,044,066 JPY (9834 ± 3808 US$)/24 weeks per treated patient in the bDMARDs group and 75,860 JPY (715 ± 155 US$)/24 weeks in the MTX group (US$ 1 = 106.16 JPY in February 2018). Two patients developed adverse drug reactions: ETN was interrupted for 3 weeks in 1 patient due to bacterial pneumonia, and the other patient in the MTX group needed 2 blood analyses due to liver dysfunction. The costs to treat these adverse reactions were excluded from this study, in line with previous study protocols [8, 9].

The mean total actual health care costs were more than tenfold higher in the bDMARDs group than in the MTX group. Furthermore, the mean cost per NNT in the bDMARDs group was 2.6-fold higher than in the MTX group based on DAS28-ESR or 1.6-fold higher based on CDAI. Although TCZ and ETN showed the lower mean costs per NNT, there was no significant difference in the mean cost per NNT between the biologic agents.

### Adjusted NNT

Through logistic regression analysis using DAS28-ESR remission rate as an objective variable, the OR of the bDMARDs group compared with the control group was 2.38 (95% confidence interval [CI] 1.02–5.54), whereas that of the MTX group was 1.61 (95% CI 0.98–3.02). On the other hand, logistic regression analysis using CDAI remission rate as an objective variable revealed ORs of 3.24 (95% CI 1.31–8.01) in the bDMARDs group and 1.72 (95% CI 0.87–3.37) in the MTX group (Table 3). We estimated the number of remissions in the bDMARDs and MTX groups as being equal to the calculated ORs, which were adjusted for bias in terms of the patients’ backgrounds (Table 4). The number of remissions according to DAS28-ESR were estimated to be 43 in the bDMARDs group and 47 in the MTX group. Correspondingly, the numbers of remissions according to CDAI were estimated to be 26 and 21.

**Table 3:** Logistic regression analysis using DAS28-ESR and CDAI remission rates as objective variables

Factors	Odds ratio (95% CI)
bDMARDs (compared with control group)	2.38 (1.02–5.54)
MTX (compared with control group)	1.61 (0.86–3.02)
Age	1.00 (0.98–1.04)
DAS28-ESR at baseline	0.81 (0.60–1.11)
Sex (male)	2.12 (0.86–5.24)
Steinbrocker stage	0.55 (0.21–1.48)
Disease duration	1.02 (0.95–1.09)
bDMARDs (compared with control group)	3.24 (0.21–8.88)
MTX (compared with control group)	1.72 (0.87–3.37)
Age	0.99 (0.96–1.02)
CDAI at baseline	0.97 (0.93–1.02)
Sex (male)	0.87 (0.36–2.07)
Steinbrocker stage	0.31 (0.11–0.92)
Disease duration	1.01 (0.95–1.08)

*bDMARDs* biologic disease-modifying anti-rheumatic drugs, *CDAI* clinical disease activity index, *CI* confidence interval, *DAS28-ESR* disease activity score in 28 joints using erythrocyte sedimentation rate, *MTX* methotrexate

**Table 4:** Adjusted number of patient remissions in DAS28-ESR and CDAI and calculated NNT

DAS28-ESR	N	Adjusted patient number of remissions	Remission rate	ARR	NNT	Mean cost per adjusted NNT^a^
bDMARDs	55	43	0.782	0.175	5.71	56,152
MTX	66	47	0.712	0.105	9.52	6807

CDAI	N	Adjusted patient number of remissions	Remission rate	ARR	NNT	Mean cost per adjusted NNT*
bDMARDs	55	26	0.473	0.259	3.86	37,959
MTX	66	21	0.318	0.104	9.62	6878

*bDMARDS* biologic disease-modifying anti-rheumatic drugs, *ARR* absolute risk reduction, *CDAI* clinical disease activity index, *DAS28-ESR* disease activity score in 28 joints using erythrocyte sedimentation rate, *MTX* methotrexate, *NNT* number needed to treat

^a^US$/24 weeks/patient

Using the adjusted remission rate in the bDMARDs and MTX groups, the adjusted NNT was calculated as 5.71 based on DAS28-ESR and 3.86 based on CDAI in the bDMARDs group and 9.52 and 9.62, respectively, in the MTX group. The adjusted NNT was lower in the bDMARDs group than in the MTX group based on both DAS28-ESR and CDAI. However, the mean costs per adjusted NNT based on both DAS28-ESR and CDAI were lower in the MTX group than in the bDMARDs group (Table 4).

## Discussion

In this study, the NNTs of bDMARDs in patients with RA were 5- to eightfold lower than those of MTX. However, the mean total actual health care costs were much higher in the bDMARDs group than in the MTX group. Mean costs per NNT based on both DAS28-ESR and CDAI were higher in the bDMARDs group than in the MTX group. This means that although the effectiveness of bDMARDs was much better than that of MTX, the cost-effectiveness of MTX was superior to that of bDMARDs from the standpoint of NNT and total actual health care cost.

Some economic measures can be used to assess the cost-effectiveness of drugs, such as the incremental cost effectiveness ratio, quality-adjusted life-years, and NNT. As mentioned earlier, NNT is not itself a true economic indicator of cost-effectiveness, but an indicator to assess the effectiveness of therapeutic drugs. However, because NNT is easy to understand and calculate, it is used to assess the cost-related effectiveness of drugs. Several studies have examined the cost-effectiveness of bDMARDs in RA using NNT. Batticciotto et al. [8] investigated NNT of monotherapy with ADA, ETN, certolizumab pegol, or TCZ in patients intolerant to MTX. They reported that TCZ had the lowest NNT and the lowest costs per NNT of all other agents, which was similar to the results of the present study. Maurizio et al. [9] compared NNT between TCZ plus MTX and ABT plus MTX in patients previously treated with MTX and concluded that TCZ was superior to ABT in terms of NNT. To our knowledge, however, no previous studies have compared bDMARDs with MTX in terms of NNT or cost per NNT.

Although simple comparison is difficult because each country has own health insurance system, several studies have reported the cost-effectiveness of bDMARDs in the treatment of RA. Hyon et al. [15] compared five monotherapies for the patients with MTX-naïve; ETN, MTX, leflunomide, SASP, and no second line agents. They reported that the costs of combination therapy of ETN plus MTX were 1.5-fold higher than those of MTX monotherapy, and MTX is cost effective choice for MTX-naïve RA patients, which is similar as our findings. Joseph et al. [16] reported that the 5 year per-patient drug costs were 4- to eightfold higher in those switched to bDMARDs versus those continued MTX. They also reported that total costs per patient were 3 to 4 times higher when adding or switching to bDMARDs than when continuing MTX. The difference of total costs between bDMARDs with MTX was smaller than our study. MTX group in this study included both oral and subcutaneous MTX, which may lead to relatively higher costs in MTX group. Because both studies calculated the costs based on simulations, they may have underestimated the actual costs. In our study, the mean costs were more than tenfold higher in the bDMARDs group than in the MTX group. We investigated the total actual costs based on the real-world clinical setting under the Japanese health insurance system, not based on simulations, which might be why the mean costs of bDMARDs were much higher than those of MTX.

Syngle et al. [17] noted that the mean direct medical cost varied due to several factors such as disease activity, variable number of patients, differences in medication, differences in methods of cost assessment, and differences in health care delivery systems worldwide. In the present study, although the costs of bDMARDs were more than tenfold higher than those of MTX, the mean costs per NNT of TCZ and ETN were equivalent to that of MTX. Based on our results, TCZ and ETN are cost-effective drugs close to MTX.

The adjusted NNT of bDMARDs was superior to that of MTX based on both DAS28-ESR and CDAI, which means that the effectiveness of bDMARDs was superior to that of MTX when patients’ backgrounds were adjusted to exclude bias. On the other hand, the costs per adjusted NNT in the bDMARDs group were eightfold higher than those of the MTX group based on DAS28-ESR and fivefold higher based on CDAI. Given that the total health care cost of the bDMARDs group was more than tenfold higher than that of the MTX group, the difference in the mean costs per adjusted NNT between the bDMARDs and MTX groups was smaller than in the mean costs per non-adjusted NNT. However, the mean cost per adjusted NNT of MTX were still superior to those of bDMARDs.

The results of this study indicate that although the effectiveness of bDMARDs was better than that of MTX, the cost-effectiveness of MTX was superior in terms of the adjusted NNT and the total costs under Japanese health insurance. Therefore, considering the costs of therapeutic drugs, we recommend MTX for first-line appropriate use in patients with RA who have no complication such as chronic kidney disease, interstitial pneumonia, or liver dysfunction that prevent the use of MTX. If the treatment target is not achieved with MTX, bDMARDs should be added immediately from the economic viewpoint, similar to the EULAR recommendations on the management of RA [5].

There are some limitations to this study. First, the placebo group should be set as controls in this type of study, however, it was difficult to assign some patients to truly placebo group because this study based on real-world clinical setting, therefore the patients who administered csDMARDs without bDMARDs or MTX was set as the control group in this study. Second, due to the small number of patients involved in this single center study, bias might exist in terms of medication selection. Particularly in the bDMARDs group, two or three specific agents were frequently used as the first bDMARDs. Especially, GLM is used for relatively higher aged patients in our hospital, in addition, 11 out of 16 patients administered with 100 mg of GLM, so the mean total health care costs in the patients who administered GLM was much higher than the other patients. Third, bio-similar of bDMARDs was not used in our hospital during the observational period in this study. The difference of total health care cost between MTX and bDMARDs will be smaller if bio-similar of bDMARDs is used because the price of bio-similar of bDMARDs are much lower than original bDMARDs. Regarding to oral medicines, the patients receive the prescript oral drugs from out-of-hospital pharmacies in principle in Japan, and pharmacies can decide whether to prescribe original or generic drugs. We did not know that the patients were prescribed original or generic drug from out-of-hospital pharmacy from the medical system of our hospital. Therefore, we calculated the costs of oral medicines assuming the prescribed drugs are all original drugs. Fourth, because the initial purpose of this study was to compare remission rates at 24 weeks between the bDMARDs and MTX groups, we excluded patients who switched medications within 24 weeks due to an adverse drug reaction or who did not need to change the former medication, which might have led to a higher remission rate than in previous studies. Finally, this study evaluated only the disease activities using DAS28-ESR and CDAI 24 weeks after the introduction of therapeutic drugs. Further investigation is required including the spacing or tapering of bDMARD, or the evaluation of joint destruction to compare between the MTX and bDMARDs groups.

Despite these limitations, we believe that this study is unique and clinically relevant. To our knowledge, no earlier studies have reported on the cost-effectiveness of bDMARDs versus MTX in patients with RA from the standpoint of NNT and total health care costs in the real-world clinical setting.

In conclusion, the NNT of bDMARDs in patients with RA, which is used as an indicator of the effectiveness of therapeutic drugs, was superior to that of MTX. However, the mean total actual health care costs were much higher in the bDMARDs group than in the MTX group. In addition, the mean costs per adjusted NNT based on both DAS28-ESR and CDAI were higher in the bDMARDs group than in the MTX group, suggesting that the cost-effectiveness of MTX is superior to that of bDMARDs from the standpoint of mean total actual health care costs per adjusted NNT under Japanese health insurance system.

## Data Availability

The datasets during and/or analysed during the current study available from the corresponding author on reasonable request.
